# Salacine

**Published:** 1845-10

**Authors:** 


*Salacine.*—Dr. Fenner, of the New Orleans Charity Hospital, has tested the virtues of this remedy in twenty-two cases of intermittent fever. The object of the observations was "to ascertain the virtues of salacine, and to what extent it may be relied upon as a substitute for quinine." Dr. Fenner deems his trial not sufficient to justify a report. He however remarks: "so far it appears greatly inferior to quinine. Its virtues are somewhat enhanced by combination with piperine. As the article has been very little used within the last few years, the quality may not be first rate. It is now dearer than quinine, on account of the larger doses required, but if it be found to answer as well *in any dose*, it can be made cheap, as the supply of willow bark in our country is inexhaustible. We are promised a report at a future time. We hope also before long to hear a report upon the subject from the office of the Surgeon General of the Army. The importance of the subject is great, if it be true, as reported in the New Orleans Medical Journal, from the Washington "Union," that the British Government are endeavoring to acquire a monopoly of Peruvian bark. We reiterate our hope that our readers will give it a trial, and send us a report of the results, whether they be favorable or unfavorable. Ed.

